# Supplementary material for: The Evolution and Characterization of the RNA Interference Pathways in Lophotrochozoa
Source: Genome Biol Evol. 2024 May 7;16(5):evae098. doi: 10.1093/gbe/evae098 (PMC11114477; doi:10.1093/gbe/evae098)
Supplement: evae098_Supplementary_Data [file evae098_supplementary_data.zip › Supplementary_materials.zip!/selected_miRNAs/CrGi_selected_miRNAs/CrGi_NC_047568.1_30001.pdf]

Provisional ID	:	NC_047568.1_30001
Score total	:	51.1
Score for star read(s)	:	3.9
Score for read counts	:	44
Score for mfe	:	2.3
Score for randfold	:	1.6
Score for cons. seed	:	-0.6
Total read count	:	98
Mature read count	:	90
Loop read count	:	0
Star read count	:	8

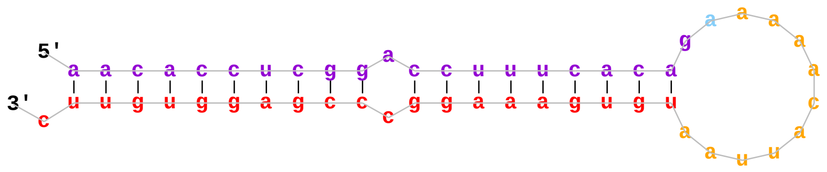

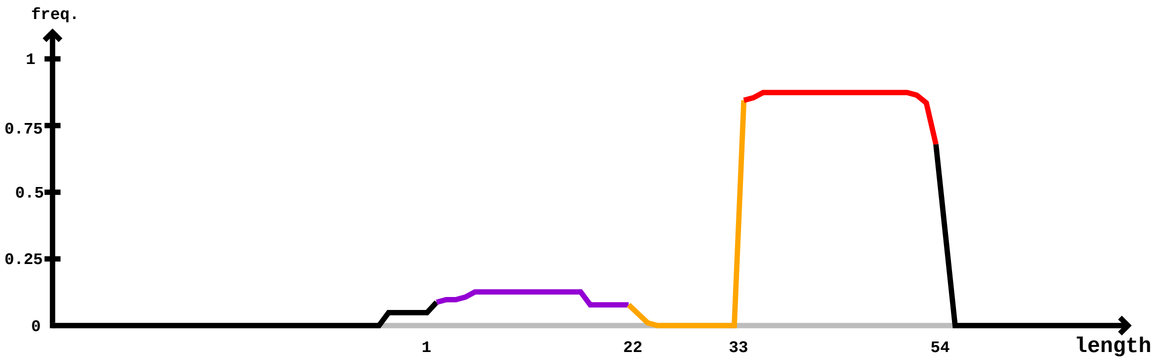


```
                                       Star                          Mature
5'- gaaauauacacaaguugugaauugucaaauucauccggaacaccucggaccuuucacagaaaaacauuaaugugaaaggcccgagguguuccggauguugucaaauuuag  -3'   obs
    gaaauauacacaaguugugaauugucaaauucauccggaacaccucggaccuuucacagaaaaacauuaaugugaaaggcccgagguguuccggauguugucaaauuuag        exp
    ........((((...))))..(((.(((...((((((((((((((((((.(((((((((...........))))))))).))))))))))))))))))))).))).....   reads   mm   sample
    ......................................aacaccucggGccuuucacag...................................................   3       1    Mt1
    ......................................................................ugugaaGggcccgaggugu.....................   1       1    Mt1
    ......................................................................ugugGaaggcccgaggugu.....................   1       1    Mt1
    ......................................................................ugugaaGggcccgagguguu....................   1       1    Mt1
    ......................................................................ugugaaaggcccgagguguu....................   5       0    Mt1
    ......................................................................ugugaGaggcccgagguguu....................   1       1    Mt1
    ......................................................................ugugaaaggcccgagguguuG...................   20      1    Mt1
    .......................................................................gugaaaggcccgagguguuG...................   1       1    Mt1
    ........................................................................ugaaaggcccgaggugu.....................   1       0    Mt1
    ........................................................................ugaaaggcccgagguguuc...................   1       0    Mt1

    ......................................................................ugugaGaggcccgaggug......................   1       1    Mt2
    ......................................................................ugugaaaggcccgGggugu.....................   1       1    Mt2
    ......................................................................ugugaaGggcccgagguguu....................   3       1    Mt2
    ......................................................................ugugaaaggcccgagguguu....................   4       0    Mt2
    ......................................................................ugugaaGggcccgagguguuc...................   1       1    Mt2
    ......................................................................ugugaaaggcccgagguguuc...................   2       0    Mt2
    ......................................................................ugugaaaggcccgagguguuG...................   22      1    Mt2

    .................................uccggaacaccuUggaccuuu........................................................   1       1    Ov2
    ..........................................ccucggGccuuucacaga..................................................   1       1    Ov2
    ......................................................................ugugaaGggcccgaggugu.....................   1       1    Ov2
    ......................................................................ugugaaaggcccgagguguuG...................   6       1    Ov2
    ......................................................................ugugaaGggcccgagguguuc...................   1       1    Ov2

    .................................uccggaacaccuUggaccuuu........................................................   3       1    Ov1
    .................................uccggaacaccucggGccuuu........................................................   1       1    Ov1
    ......................................aacaccucggGccuuucacag...................................................   1       1    Ov1
    .......................................acaccucggGccuuucacag...................................................   1       1    Ov1
    .........................................accucggGccuuucacagaa.................................................   1       1    Ov1
    ..........................................ccucggGccuuucacaga..................................................   1       1    Ov1
    ......................................................................ugugaaaggcccgagguguuc...................   1       0    Ov1
    ......................................................................ugugaaaggcccgagguguuG...................   15      1    Ov1
```

Star Mature

gaaauauacacaaguugugaauugucaaauucauccggaacaccucggaccuuucacagaaaaacauuaaugugaaaggcccgagguguuccggauguugucaaauuuag
